# Cytokines Released by Human Kidneys During Normothermic Machine Perfusion are not Associated With Early Posttransplant Outcomes

**DOI:** 10.1097/TP.0000000000005744

**Published:** 2026-05-01

**Authors:** Karim Bousnina, Robert C. Minnee, Karin Boer

**Affiliations:** 1 Division of Nephrology and Transplantation, Department of Internal Medicine, Erasmus MC Transplant Institute, Erasmus University Medical Center, Rotterdam, the Netherlands.; 2 Department of Surgery, Division of HPB and Transplant Surgery, Erasmus MC Transplant Institute, Erasmus University Medical Center, Rotterdam, the Netherlands.

## Abstract

**Figure:**
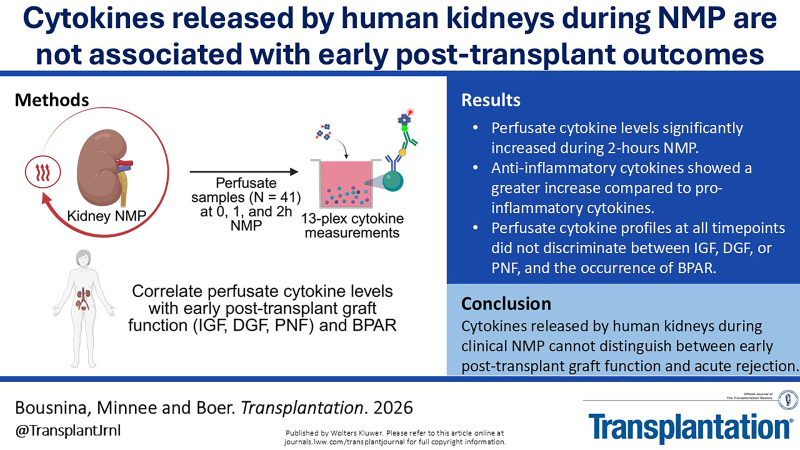

The pathophysiologic effects (including vascular injury) from deceased organ donation trigger a systemic inflammatory response, that is, a cytokine storm, that can impair graft quality at transplantation.^1^ Endothelial cells are directly exposed to these mediators and especially vulnerable to transplantation-associated injury, further damaging the allograft.^2^ Normothermic machine perfusion (NMP) offers the opportunity to evaluate kidney quality pretransplant, providing valuable insight into graft immunogenicity and injury. In this study, we analyzed perfusate cytokines and chemokines associated with (vascular) inflammation released by human kidneys during NMP and investigated their association with early posttransplant outcomes.

Kidney perfusate samples (n = 41) from a randomized controlled trial (NCT04882254, https://clinicaltrials.gov/) were collected at 0, 1, and 2 h of NMP. Cytokines were quantified with a human vascular inflammation panel kit (catlog No. 740966; LEGENDplex, BioLegend, CA) per manufacturer’s instructions and analyzed by flow cytometry. Cytokine concentrations were expressed in pg/mL after subtraction of control values derived from unused perfusate. The same control reference values were applied to all samples before log_10_ transformation. Endpoints included immediate graft function, delayed graft function (DGF; need for dialysis in the first week after transplantation), primary nonfunction (never functioning graft 3 mo after transplantation), and biopsy-proven acute rejection within the first 2 wk after transplantation. A 1-way ANOVA or Wilcoxon signed-rank test was used to test for group differences. A linear mixed-effects model was used for concentration changes over time. *P* values were Benjamini-Hochberg-adjusted, with adjusted 2-tailed *P* value of ≤0.05 considered statistically significant. Data were processed in RStudio (version 2024.04.2).

All perfusate cytokine levels significantly increased during the first hour of NMP. In the second hour, only C-C motif chemokine ligand 2, interleukin (IL)-10, IL-6, soluble CD40 ligand, soluble suppression of tumorigenicity 2, tyrosine kinase with immunoglobulin and epidermal growth factor homology domain 2, and tumor necrosis factor alpha showed a significant rise (Figure 1A). Contrary to a previous study in a porcine model,^3^ we did not observe an inflammatory milieu during perfusion. Instead, anti-inflammatory cytokine concentrations increased 2.1-fold (*P* = 0.0005) and 1.3-fold (*P* = 0.062) more than proinflammatory cytokines during the first and second hour, respectively, suggesting a more anti-inflammatory perfusion environment. Nevertheless, cytokine profiles at 1 and 2 h NMP were not associated with early posttransplant graft function (immediate graft function, DGF, and primary nonfunction) and biopsy-proven acute rejection (Figure 1B and C).

**FIGURE 1.**
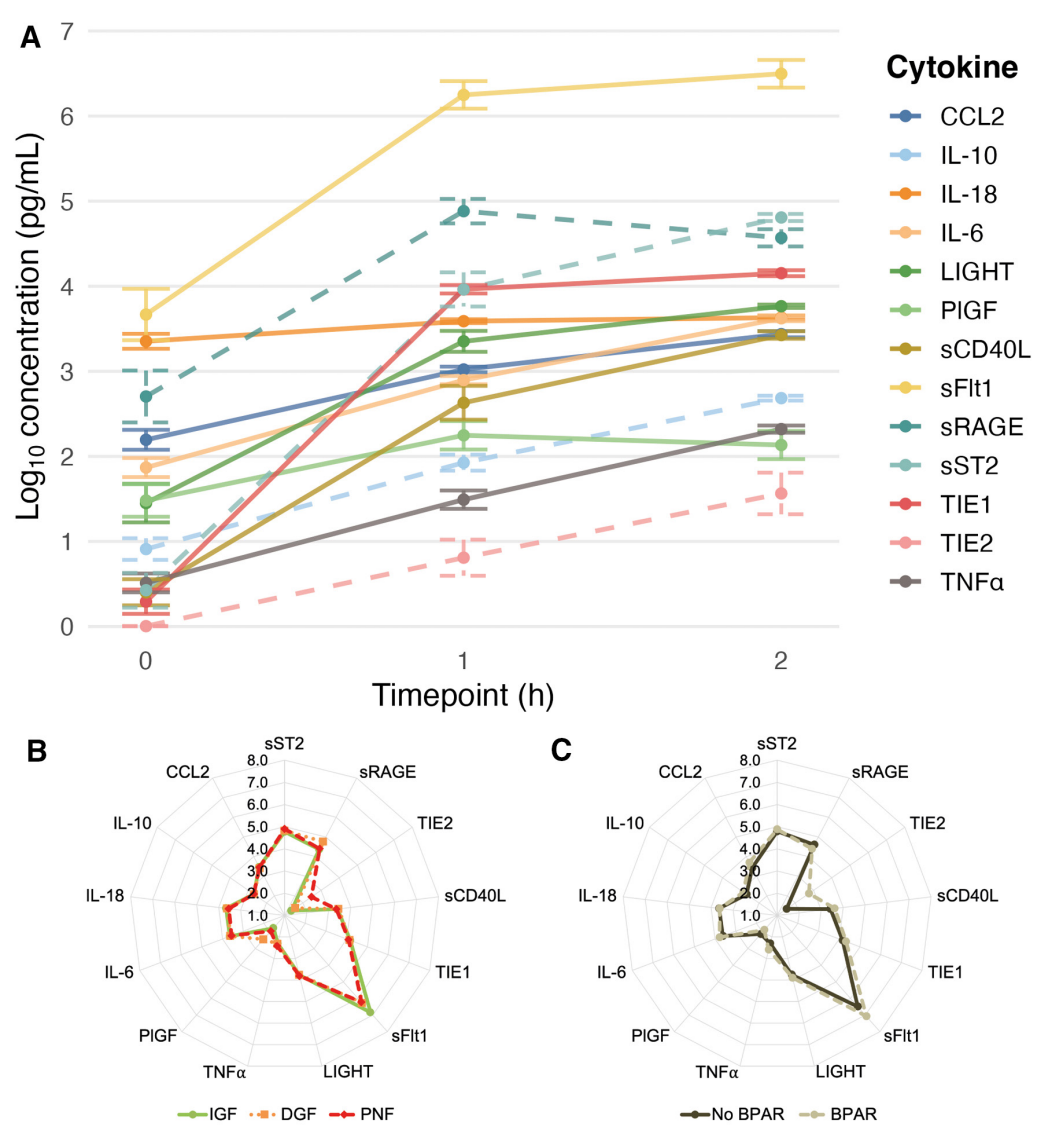
A, Mean log_10_ perfusate concentration (pg/mL) of cytokines at 0, 1, and 2 h of NMP. Solid and dashed lines indicate pro- and anti-inflammatory cytokines, respectively. Error bars represent SE. B and C, Radar chart showing the mean log_10_ concentrations (pg/mL) of 13-plex cytokine measurements in kidney perfusate samples (n = 41) after 2 h NMP (1-h NMP data not shown). Profiles are stratified for (B) posttransplant graft function (IGF [n = 16], DGF [n = 22], and PNF [n = 3]), and (C) BPAR (no BPAR [n = 35], BPAR [n = 4], and borderline [not shown, n = 2]). BPAR, biopsy-proven acute rejection (within the first 2 wk after transplantation); CCL2, C-C motif chemokine ligand 2; DGF, delayed graft function (need for dialysis in the first week after transplantation); IGF, immediate graft function; IL, interleukin; LIGHT, homologous to lymphotoxins, exhibits inducible expression, and competes with herpes simplex virus glycoprotein D for herpesvirus entry mediator, a receptor expressed by T lymphocytes; NMP, normothermic machine perfusion; proinflammatory cytokines: sCD40L, soluble CD40 ligand; PlGF, placental growth factor; PNF, primary nonfunction (never functioning graft 3 mo after transplantation); sFlt1, soluble Fms-like tyrosine kinase-1; Srage, soluble receptor for advanced glycation end-product; sST2, soluble suppression of tumorigenicity 2; TIE1, tyrosine kinase with immunoglobulin and epidermal growth factor homology domain 1; TIE2, tyrosine kinase with immunoglobulin and epidermal growth factor homology domain 2; TNFα, tumor necrosis factor alpha.

Recently, markedly higher IL-18 levels (~10-fold) were reported in NMP perfusates,^4^ which could be explained by the preceding cold storage, whereas here NMP was performed after hypothermic machine perfusion. The association between higher IL-18 levels and DGF was not confirmed in this study, which underscores that perfusate cytokines cannot yet be considered reliable standalone prognostic biomarkers. Instead, they may be more appropriately applied to evaluate (immune)modulatory interventions during NMP. Although in our study cytokine levels did not correlate with early posttransplant outcomes, it should be noted that the present study was explorative and the sample size was not powered for predictive modeling. Overall, in our study, kidneys released a broad range of cytokines, likely reflecting graft injury responses, but the inflammatory state of the kidney may be better captured using complementary approaches, such as tissue-based analyses.^5^

## ACKNOWLEDGMENTS

The authors thank Yvette den Hartog, Julia S. Slager, Yitian Fang, Marjolein Dieterich, Carla C. Baan, and Martin J. Hoogduijn for their valuable contributions to this work, including providing samples, supporting and guiding the data analysis, performing the cytokine measurements, and contributing to the research design.

## References

[1] Floerchinger B Oberhuber R Tullius SG. Effects of brain death on organ quality and transplant outcome. Transplant Rev (Orlando). 2012;26:54–59. 22459036 10.1016/j.trre.2011.10.001

[2] Cardinal H Dieudé M Hébert MJ. Endothelial dysfunction in kidney transplantation. Front Immunol. 2018;9:342152. 10.3389/fimmu.2018.01130 PMC5974048 29875776

[3] Stone JP Ball AL Critchley WR. Ex vivo normothermic perfusion induces donor-derived leukocyte mobilization and removal prior to renal transplantation. Kidney Int Rep. 2016;1:230–239. 29142927 10.1016/j.ekir.2016.07.009 PMC5678860

[4] Dumbill R Knight S Hunter J. Prolonged normothermic perfusion of the kidney prior to transplantation: a historically controlled, phase 1 cohort study. Nat Commun. 2025;16:4584–4515. 40382321 10.1038/s41467-025-59829-5 PMC12085653

[5] Li S Tejeda-Mora H Slagter JS. Cellular responses during kidney normothermic machine perfusion reflect graft outcomes. Kidney Int Rep. 2025;10:4012–4026. 41278344 10.1016/j.ekir.2025.08.017 PMC12639818

